# Complete genomes of the eukaryotic poultry parasite *Histomonas meleagridis:* linking sequence analysis with virulence / attenuation

**DOI:** 10.1186/s12864-021-08059-2

**Published:** 2021-10-21

**Authors:** Nicola Palmieri, Marcelo de Jesus Ramires, Michael Hess, Ivana Bilic

**Affiliations:** 1 Clinic for Poultry and Fish Medicine, Department for Farm Animals and Veterinary Public Health, University of Veterinary Medicine, Vienna, Austria; 2 Christian Doppler Laboratory for Innovative Poultry Vaccines (IPOV), University of Veterinary Medicine Vienna, Vienna, Austria

**Keywords:** *Histomonas meleagridis*, Virulent, Attenuated, Histomonosis, Genome, Turkey, Chicken, Virulence, Oxford Nanopore technology (ONT), Illumina, Leucine rich repeat protein

## Abstract

**Background:**

*Histomonas meleagridis* is a protozoan parasite and the causative agent of histomonosis, an important poultry disease whose significance is underlined by the absence of any treatment and prophylaxis. The recent successful in vitro attenuation of the parasite urges questions about the underlying mechanisms.

**Results:**

Whole genome sequence data from a virulent and an attenuated strain originating from the same parental lineage of *H. meleagridis* were recruited using Oxford Nanopore Technology (ONT) and Illumina platforms, which were combined to generate megabase-sized contigs with high base-level accuracy. Inspecting the genomes for differences identified two substantial deletions within a coding sequence of the attenuated strain. Additionally, one single nucleotide polymorphism (SNP) and indel targeting coding sequences caused the formation of premature stop codons, which resulted in the truncation of two genes in the attenuated strain. Furthermore, the genome of *H. meleagridis* was used for characterizing protein classes of clinical relevance for parasitic protists. The comparative analysis with the genomes of *Trichomonas vaginalis*, *Tritrichomonas foetus* and *Entamoeba histolytica* identified ~ 2700 lineage-specific gene losses and 9 gene family expansions in the *H. meleagridis* lineage.

**Conclusions:**

Taken as a whole, the obtained data provide the first hints to understand the molecular basis of attenuation in *H. meleagridis* and constitute a genomics platform for future research on this important poultry pathogen.

**Supplementary Information:**

The online version contains supplementary material available at 10.1186/s12864-021-08059-2.

## Background

*Histomonas meleagridis* is a flagellated extracellular poultry parasite of the order Tritrichomonadida [1]. It causes histomonosis (syn. Histomoniasis, blackhead disease, infectious typhlohepatitis) an important disease of gallinaceous birds, especially in turkeys and chickens [2]. The disease can be very devastating in turkeys, in which the parasite causes serious ceacal lesions and liver necrosis that can lead up to 100% mortality [3]. In chickens, histomonosis is less severe and the infection is generally confined to the caeca [4]. Histomonosis and the parasite are known for more than 100 years, which in the second half of the twentieth century led to the introduction of effective prophylactic and chemotherapeutic drugs with the almost disappearance of the disease [5]. The situation drastically changed at the beginning of the present century, when these active compounds were banned as a result of amendments in drug legislation in the European Union and the USA and histomonosis reappeared with most severe consequences in turkeys [6]. In addition, histomonosis became more prevalent in chickens aided by the tendency to increase free-range farming, enabling bird’s access to the parasite [4]. Up to now, only a prototype live vaccine, based on an in vitro attenuated strain has been shown to be effective against infection with *H. meleagridis* [7]. Nonetheless, the vaccine is not yet commercially available; in an attempt to prevent mortality, veterinarians can only rely on the implementation of the proper flock management and the very early administration of the off-licensed aminoglycoside antibiotic paromomycin [5, 6]. Succeeding decades without any research, the ban of effective drugs and the reappearance of histomonosis, resurrected the investigations on the parasite and the disease itself. The majority of early molecular studies focused on the phylogenetic positioning of *H. meleagridis*, with just a handful of research papers reporting genetic information on few protein coding genes [4]. Only recently, omics-based approaches greatly improved the molecular knowledge on this important poultry parasite, by supplying de novo transcriptome database, as well as the results of proteome and exoproteome analyses [8–11]. However, up to now, the complete genome of *H. meleagridis* is not available.

Molecular investigations of *H. meleagridis* depend on its in vitro culture, in which the parasite can be propagated only in the presence of bacteria [4]. In the laboratory, *H. meleagridis* is usually propagated through an in vitro xenic culture, together with turkey or chicken caecal flora. The culture is set up by inoculating an intestinal content of a bird suffering from histomonosis into a suitable cell culture media. In order to standardize this procedure and to obtain a more defined culture, a “clonal or mono-eukaryotic” culture was established by transferring a single protozoan cell to fresh medium via micromanipulation [12]. Further improvement of such culture was the replacement of ill-defined caecal bacterial flora by a single *Escherichia coli* strain, without compromising the virulence of the parasite [13].

In the present study, we sequenced the genomes of two *H. meleagridis* strains, a monoxenic clonal virulent and an attenuated strain, both of which can be traced to the same single cell. In addition to the novelty of the genome sequence, we analyzed both genomes in respect to their differing virulent phenotypes. Identified mutations suggested potential virulence targets that were inactivated or modified in the course of attenuation.

## Results

### Genome assembly and annotation

The whole genomes of two *H. meleagridis* strains – a virulent and an attenuated strain – were sequenced and annotated with the goals of expanding genomic information on this important poultry pathogen and investigating the genomic basis of attenuation. The strains used for whole genome sequencing originate from a single parasitic cell that was transferred via micromanipulation from the initial culture into the fresh suitable medium, establishing a so called “mono-eukaryotic clonal culture”. Further prolonged in vitro cultivation resulted in the attenuation of the parasite. As aliquots of every cultivation passage were cryopreserved, the retracement to the original virulent *H. meleagridis* was possible.

**Fig. 1:**
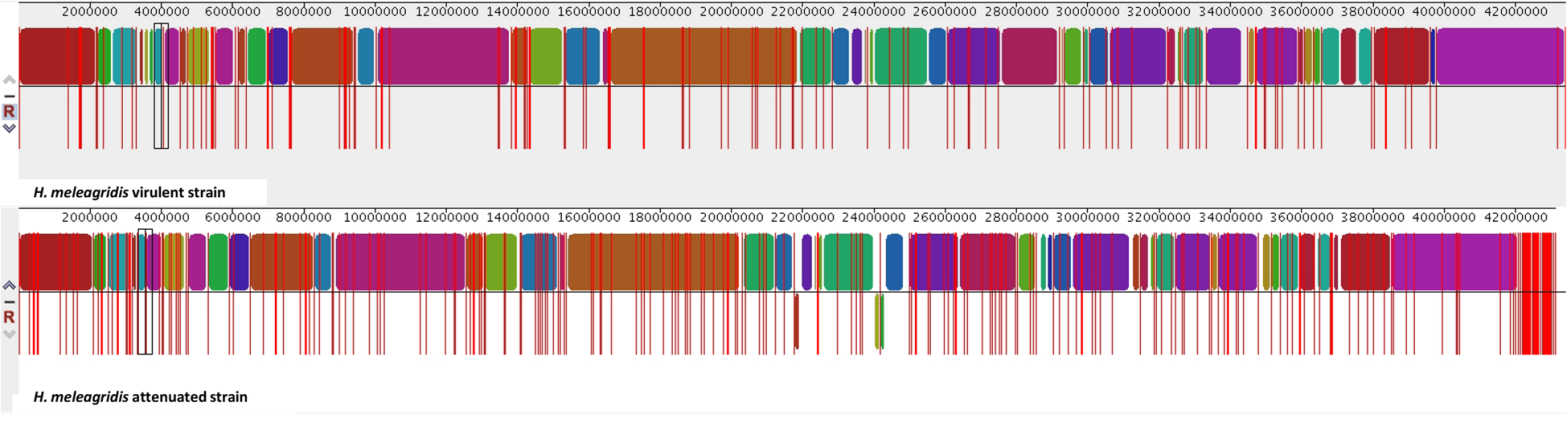
Alignment of two *H. meleagridis* genomes by MAUVE displaying the orthologous blocks between the virulent strain (top track) and the attenuated strain (bottom track). Synteny blocks between the two strains are marked with the same color, three small inverted blocks are apparent in the attenuated strain as blocks that are drawn in downward orientation

In order to achieve megabase-sized contigs with high base-level accuracy, the genomes of both strains were assembled using a combination of ONT long reads and Illumina short reads. The genome of *H. meleagridis* is 43 Mb in size, is GC poor (28%) and contains about ~ 11,000 genes, with little variation between strains (Table 1). Introns are scarce, with 5.2–5.7% of genes containing at maximum one intron. Repetitive regions account for about ~ 2% of the genome in both strains and include microsatellites (1.63–1.75%), low complexity sequences (0.51–0.53%) and different classes of transposable elements (Table S1). The overall genome duplication level in *H. meleagridis* was computed directly as the fraction of the genome that is duplicated instead of inferring this figure by indirect methods (i.e. [14]). To calculate this, we aligned the contigs to themselves with nucmer and extracted homologous regions with the show-coords tool [15]. This resulted in a duplication level of 20% for both strains. To evaluate the accuracy of the assemblies we employed three metrics: i) base-level accuracy, ii) presence of eukaryotic core genes and iii) completeness of gene models. Base-level accuracy was computed by aligning the assembly of each strain to another assembly generated using only Illumina read and computing the average genome similarity with the dnadiff [15] tool, since the accuracy from Illumina reads is higher compared to the ONT reads [16]. Using this approach, we obtained 99.82% accuracy for the virulent strain and 99.79% for the attenuated strain. Next, we computed the percentage of eukaryotic core genes from the CEGMA database using BLAST. We opted to use CEGMA for this analysis rather than the newer tool BUSCO, as the latter program works at best with a lineage-specific genes database, which is not available for Parabasalia [17]. Out of total 458 core genes we detected 395 (86%) in the virulent strain and 395 (86%) in the attenuated strain, with 100% overlap between the two datasets, which is in accordance with the high similarity in gene content between strains. Last, we evaluated the completeness of gene models by classifying the CDS into complete (having stop and start codon) or partial (having only stop or only start) and found that 100% of the genes have complete CDS in both strains, underlining the high quality of the assemblies. To get an overview of genomic rearrangements for both assemblies, we aligned the contigs from the virulent strain to the attenuated strain using MAUVE [18]. The alignment shows high colinearity between the two strains, no genomic rearrangements within contigs and three inverted contigs in the attenuated strain (Fig. 1). Overall, we observed higher genome fragmentation for the attenuated strain, concordant with the higher number of contigs (Table 1). Finally, since the in vitro growth of the parasite is strictly dependent on the presence of *E. coli* in the medium, we characterized the *E. coli* genome sequence accompanied with both strains [13]. Both strains were associated to the *E. coli* DH5α strain. For the virulent strain, the *E. coli* genome sequence is located on contig 42, which is 4,509,917 bp long and contains 4205 genes. For the attenuated strain, the *E. coli* genome sequence is located on contig 59, is 4,512,252 bp long and contains 4215 genes, with 99.98% similarity between the two *E. coli* sequences.

**Table 1:** Genome statistics for *H. meleagridis* virulent and attenuated strains

genomic feature	virulent strain	attenuated strain
number of contigs	187	281
Largest contig (bp)	3,384,422	1,157,039
Total length (bp)	43,414,808	43,133,364
N50 (bp)	673,467	314,021
GC content	28.66%	28.77%
Genes	11,119	11,137
Transcripts	11,119	11,137
Exons	11,692	11,775
Introns	573	638
Retroelements	32	23
Retroelements (bp)	5358 (0.01%)	2568 (0.01%)
DNA transposons	226	235
DNA transposons (bp)	96,463 (0.22%)	100,787 (0.23%)
Total interspersed repeats (bp)	101,821(0.23%)	103,355 bp (0.24%)
Simple repeats	14,135	13,433
Simple repeats (bp)	761,166 (1.75%)	704,125 (1.63%)
Low complexity regions	4320	4426
Low complexity regions (bp)	220,103 (0.51%)	228,092 (0.53%)

### Differences in gene content between strains

**Fig. 2:**
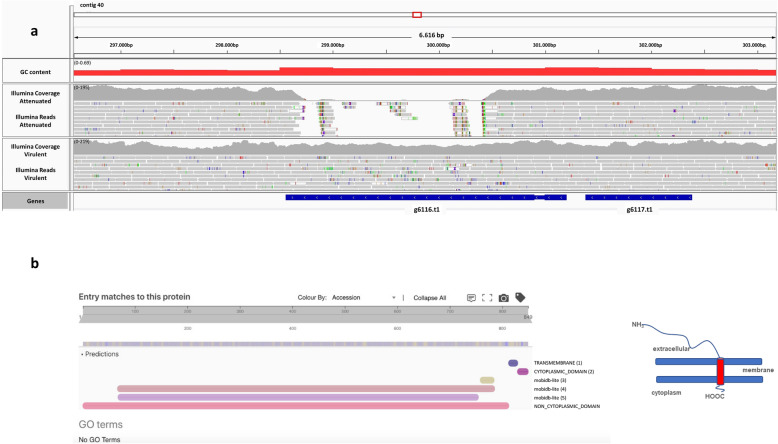
Characterization of the deletion in g6116 _vir_: (**a**) Read coverage from Illumina data of both strains; (**b**) Domain prediction from InterProScan on the g6116_vir_ protein. In the section ‘Entry matches to this protein’, six domains are predicted across the protein sequence, including a large non cytoplasmic domain, a small terminal transmembrane domain followed by a small cytoplasmic domain. This suggests that the protein is anchored to the membrane and has a long N-terminal extrusion that projects into the extracellular space (see the simplified cartoon)

**Fig. 3:**
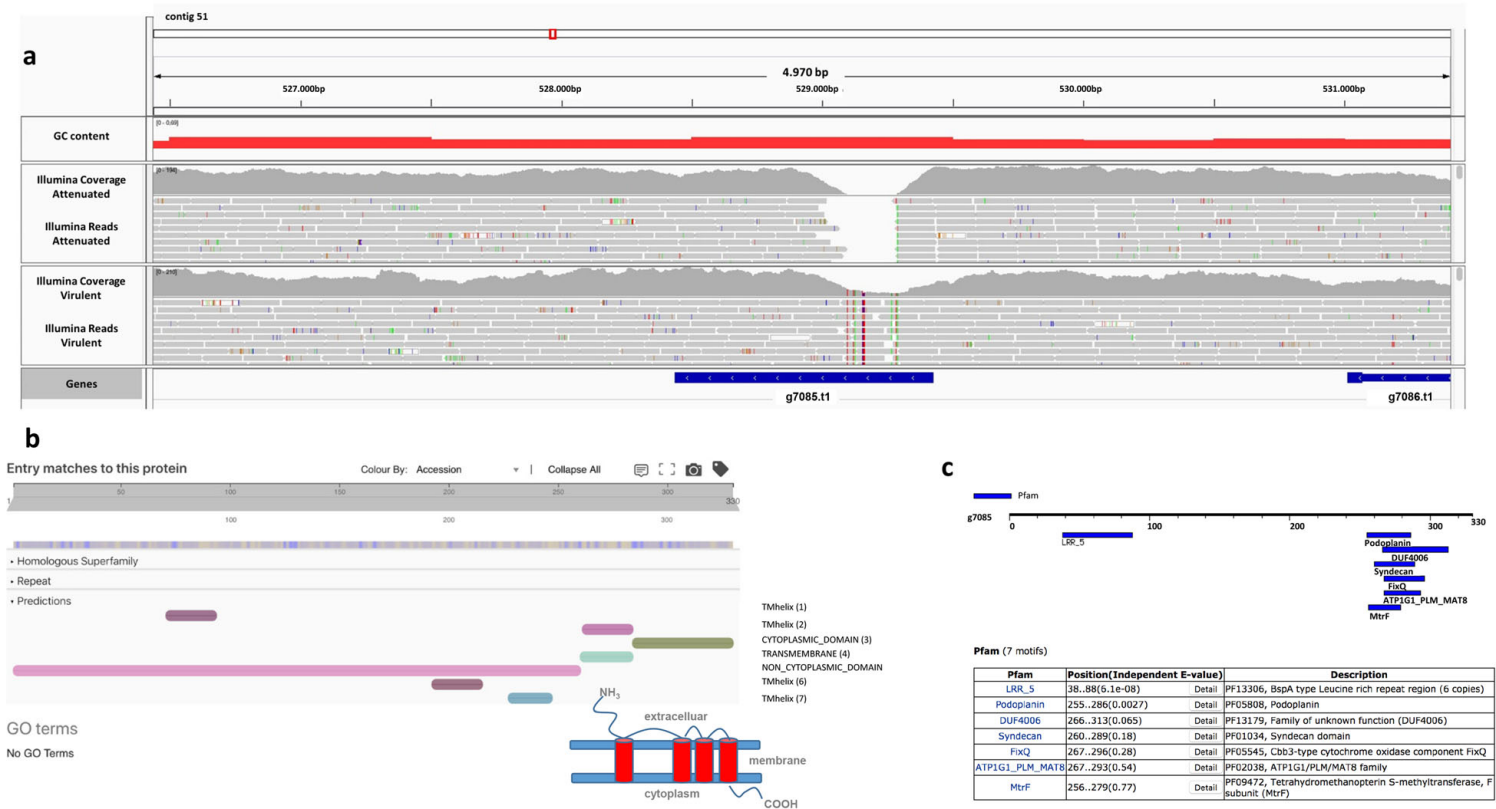
Characterization of the deletion in g7085_vir_: (**a**) Read coverage from Illumina data of both strains for the g7085_vir_; (**b**) Domain prediction from InterProScan on the g7085_vir_ protein. In the section ‘Entry matches to this protein’, seven domains are predicted across the protein sequence, including four transmembrane helices, suggesting a multi-pass transmembrane topology with a long N-terminal extracellular tail (see the simplified cartoon) (**c**) Pfam motif prediction showing the location of the motifs, note the presence of the BspA type motifs at the N terminus

In order to find evidence for putative strain-specific losses/deletions or gene duplications that might have occurred during the prolonged passaging of *H. meleagridis*, we compared gene content between the two sequenced strains. For this analysis, we clustered the protein sequences of both strains using OMA [19] to detect gene families. A total of 11,119 genes from the virulent strain and 11,137 genes from the attenuated strain were clustered into 10,063 gene families. From this dataset we extracted 400 genes that were found only in the attenuated strain and 281 of them aligned to the virulent strain using Exonerate [20] (parameters –model protein2genome –percent 90 –bestn 1), indicating that these genes simply represent missing annotations in the attenuated strain. Of the remaining 119 genes, five of them (g8917_att_ ➔ g8921_att_ on contig 49) had no support from Illumina reads in the virulent strain, pointing to a deletion in the virulent strain. As these genes did not have any marked functional annotation, additional BLAST searches were conducted, which revealed that these five genes belonged to a phage contamination located on a chimeric contig. Using the same approach, we extracted 335 genes that were found only in the virulent strain and found that 234 of them aligned the attenuated strain using Exonerate. Of the remaining 101 genes, 99 of them had full support from Illumina reads, indicating missing annotations, while two of them (g6116_vir_ on contig 40 and g7085_vir_ on contig 51) had only partial support from Illumina reads in the attenuated strain (Fig. 2a, Fig. 3a), pointing to a deletion that occurred in the attenuated strain. The g6116_vir_ gene encodes for a hypothetical protein with transmembrane domain towards the C-terminus as identified by InterProScan [21] (Fig. 2b). The g7085_vir_ encodes a leucine-rich repeat (LRR) domain-containing protein with four predicted transmembrane helices (Fig. 3b). As LRR proteins of the BspA family are involved in virulence in trichomonads [22], we looked at the presence of BspA motifs in this protein with the online tool MOTIF (genome.jp/tools/motif/) and identified a single BspA motif at positions 38 ➔ 88 (Fig. 3c).

### Confirmation of gene deletions

In order to confirm deletions of g6116_vir_ and g7085_vir_ in the attenuated strain and narrow the time point, i.e. passage number, when the deletion occurred, we tested a range of different passages for the presence/absence of loci by conventional and real-time PCR (Table 2). We confirmed the g6116_vir_ deletion in the attenuated strain, whereas the deletion of the g7085_vir_ could not be experimentally validated (Table 2). The analysis showed that the deletion of the g6116_vir_ occurred already in the xenic background between the passage 83 and 145. In addition, testing low and high passages of other unrelated *H. meleagridis* strains, grown as xenic clonal cultures, demonstrated that g6116_vir_ did not change and remained as wild type throughout the in vitro cultivation (Table 2, Fig. S1).

**Table 2:** Confirmation of variants. The confirmation of deletions was performed by both real-time PCR and conventional PCR with Sanger sequencing of PCR products. The verification of indel and SNP, both causing premature stop codon, used conventional PCR coupled with Sanger sequencing of PCR products. 18S real-time PCR [23] was performed to control for the presence/amount of *H. meleagridis* DNA

strain	passage number	g6116_**vir**_ conv. PCR	g6116_**vir**_ real-time PCR	18S real-time PCR^**a**^	g7085_**vir**_ (conv PCR, real-time PCR)	indel (AGC Kinase)	SNP (LRR)
**monoxenic 2922-C6/04/DH5 α**	**28p**	+	+ (Ct 23.68)	+ (Ct16.29)	+	wt^c^	wt
	**290p**	–	- (no Ct)	+ (Ct18.70)	+	premature stop	premature stop
**xenic 2922-C6/04**	**13p**	+	+ (27.59)	+ (Ct19.87)	+	wt	wt
	**51p**	+	+ (Ct29.93)	+ (Ct17.10)	+	wt	wt
	**83p**	+	+ (Ct 32.25)	+ (Ct21.49)	+	wt	wt
	**145p**	–	- (no Ct)	+ (Ct19.47)	+	premature stop	wt
	**237p**	–	- (no Ct)	+ (Ct18.05)	+	premature stop	premature stop
	**292p**	–	- (no Ct)	+ (Ct16.23)	+	premature stop	premature stop
**xenic 2877-C3/05**	**22p**	+	+ (Ct25.57)	+ (Ct18.31)	n.d.^b^	wt	wt
**xenic 8175-C7/06**	**21p**	+	+ (Ct26.02)	+ (Ct18.07)	n.d.	wt	wt
**xenic 13,250-C20/10**	**24p**	+	+ (Ct24.83)	+ (Ct17.27)	n.d.	wt	wt
	**316p**	+	+ (Ct22.91)	+ (Ct16.13)	n.d.	wt	wt

^a^ [23]

^b^*n.d.* not done

^c^*wt* wild type

### Variants identification between strains

**Fig. 4:**
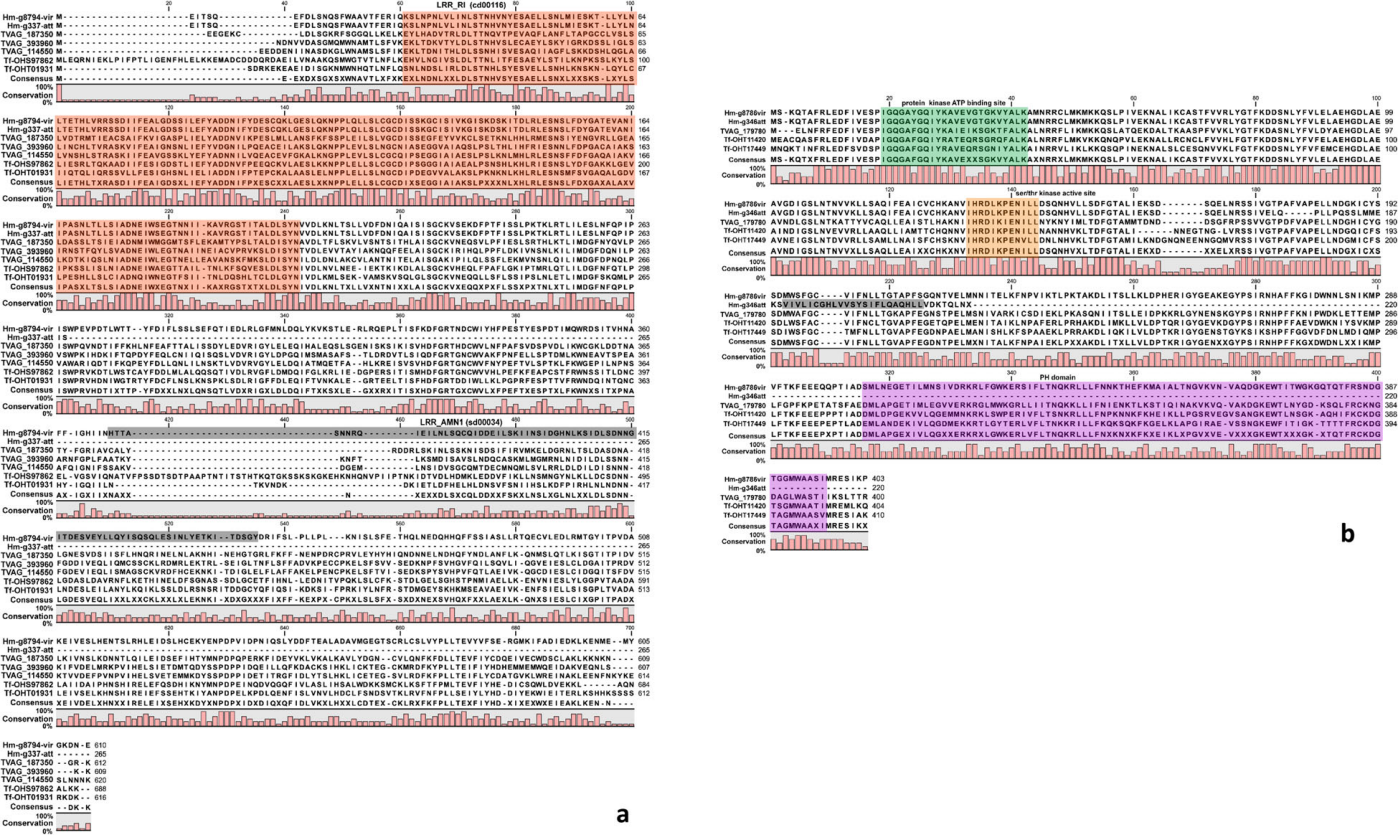
Multiple amino acid alignment between the two *H. meleagridis* strains and the orthologs in *T. vaginalis* and *T. foetus* for genes displaying the truncation of the protein sequence in the attenuated strain. (**a**) g8794_vir_/g337_att_ with SNP and (**b**) g8786_vir_/ g346_att_ with indel. Major protein domains are labeled. In the (**a**) orange box shows Leucine-rich repeats (LRRs), ribonuclease inhibitor (RI)-like subfamily conserved domain (LRR_RI (cd116)) and the grey box leucine-rich repeats, antagonist of mitotic exit network protein 1-like subfamily structural motif (LRR_AMN1 (sd00034)). In the (**b**) the green box designates protein kinase ATP binding site, orange box serine/threonine kinase active site, the purple a pleckstrin homology domain (PH) and the grey box a transmembrane domain in g346_att_ formed due to a frameshift

Next, we looked at SNPs and indels between stains to find variants affecting the coding regions (non-synonymous and missense mutations), as these could be easily correlated with a change of gene function that occurred during the attenuation process. The critical step in this analysis is to distinguish true variants from sequencing errors introduced by the ONT reads [24]. After aligning the genome of the attenuated to the virulent strain with nucmer, we identified an initial set of 17,170 SNPs using the show-snps tool [15]. After removing sequencing errors (see Methods, Identification of variants between strains), we obtained a filtered set of 68 homozygous SNPs and 2933 heterozygous SNPs. We considered only homozygous SNPs for following analyses as the true genotype cannot be determined for heterozygous SNPs. Of the 68 homozygous SNPs, 17 were located within coding regions (Table 3): of these, 4 were synonymous, 12 non-synonymous and 1 caused a premature stop codon in a protein of the attenuated strain (g337_att_) (Fig. 4a). Using the local BLAST search against transcriptomes of the virulent and attenuated strain [8] it became evident that both strains express this gene, with the transcript from the attenuated gene displaying the SNP. As the gene g337_att_ encodes for a leucine-rich (LRR) repeat protein, again we looked at the presence of BspA domains in the non-truncated version of g337_att_ of the virulent strain (g8794_vir_) but did not find any. Both versions, a complete (g8794_vir_) and a truncated (g337_att_) contain LRR_RI superfamily domain (sd00116) located in the N-terminal part, however the truncated version lacked the second LRR domain (LRR_AMN1 (sd00034)) (Fig. 4a). The characterization of the truncation with PROVEAN tool (Protein Variation Effect Analyzer) [25] marked the event as deleterious for the protein function (Table 3). The remaining non-synonymous SNPs were also characterized with PROVEAN; 2 out of 12 were marked as deleterious, although both of them did not affect functional domains. Indels were detected from the alignment of the attenuated to the virulent strain using the show-snps tool [15] with the indel option selected. The initial unfiltered set contained 21,822 indels. After extracting homozygous indels supported by the Illumina reads we obtained 2 true indels: the first was an insertion of an A located in a non-coding region: a microsatellite located at position 151,264_vir_ of contig 33, which we disregarded as common microsatellite variation. However, the second was a deletion of a G located in the coding region of gene g346_att_ which generated a frameshift introducing a premature stop codon. Alignment of g346_att_ together with the corresponding orthologs in the virulent strain (g8786_vir_) and the other sequenced Parabasalia species confirmed that the truncation of g346_att_ is specific to the attenuated strain (Fig. 4b). Both g346_att_ and g8786_vir_ encode for an AGC family protein kinase, as evident by the presence of a protein kinase ATP binding site and the serine/threonine kinase site in both coding sequences (Fig. 4b). However, due to the indel in g346_att_ coding sequence, the pleckstrin homology domain located in the C-terminal part of g8786_vir_ and other Parabasalid orthologues was lost in g346_att_ (Fig. 4b). The PROVEAN tool characterization of the mutations that were caused by the frameshift, such as the exchange 38 amino acids and the truncation, labelled the indel event as deleterious for the protein. Interestingly, the complete exchange of the last 38 amino acids before the premature stop codon, produced a short transmembrane domain at the C-terminus of g346_att_ (Fig. 4b). It seems that neither of the strains expressed this gene during in vitro growth as no transcript hit was detected using local BLAST against the transcriptome [8].

**Table 3:** List of homozygous SNPs located within the coding region. Position of the SNP and CDS coordinates refer to the virulent strain

contig (virulent strain)	SNP position	CDS start	CDS end	CDS strand	gene ID virulent strain	gene ID attenuated strain	functional annotation	SNP type	PROVEAN prediction (threshold ≤ −2.5)
contig_1	808,350	807,890	809,134	–	g222	g1540	Clan MG, family M24, aminopeptidase P-like metallopeptidase	non synonymous missense T262R	neutral (score − 0.566)
contig_4	907,239	906,962	907,699	+	g6022	g2624	unknown; domain superfamily: PRK05901-RNA polymerase sigma factor, provisional	non synonymous missense D93V	deleterious
contig_9	56,933	56,370	59,399	+	g9263	g1249	Rho-GEF	non synonymous missense M188I	neutral (score 1.600)
contig_9	754,604	754,419	755,309	–	g9462	g9315	Ser/Thr protein phosphatase	non synonymous missense F236V	neutral (score 0.252)
contig_24	8498	8153	9325	+	g3899	g1904	guanidine-nucleotide exchange factor domain containing protein	non synonymous missense P116S	neutral (score 0.717)
contig_24	135,940	135,268	135,959	+	g3944	g1860	CAMK family serine/threonine-protein kinase 25-like	non synonymous missense M258K	deleterious (score − 2906.0)
contig_30	608,903	608,341	611,167	+	g4054	g10480	alpha amylase domain-containing protein	non synonymous missense S188F	neutral (score − 0.233)
contig_39	136,516	136,175	136,771	+	g5742	g4148	transposable element Tcb2 transposase	synonymous	n.d.*
contig_46	126,968	124,401	127,370	–	g6691	g4310	BspA family leucine-rich repeat surface protein	non synonymous missense E135K	neutral (score 0.067)
contig_51	970,026	967,493	970,771	+	g7214	g10757	Major Facilitator Superfamily transporter	non synonymous missense K845M	neutral (score − 1.744)
contig_55	233,376	232,271	233,587	+	g7655	g5929	Myb-like DNA-binding domain containing protein	non synonymous missense R369M	neutral (score − 0.712)
contig_68	549,886	546,983	550,105	+	g8542	g6202	unknown	synonymous	n.d.
contig_68	639,193	639,152	640,081	–	g8565	g6179	hypothetical protein	non synonymous missense M297L	neutral (score − 1.0)
contig_72	57,296	56,499	58,331	+	g8794	g337	leucine rich repeat containing protein	non synonymous nonsense W266del	deleterious (score − 56.252)
contig_82	121,399	120,392	121,525	+	g9013	g1396	type IIB DNA topoisomerase family protein	synonymous	n.d.
contig_118	307,118	306,262	307,380	–	g931	g3679	POC1 centriolar protein homolog B	non synonymous missense P88R	neutral (score 0.655)
scaffold_61	3,064,020	3,064,015	3,066,186	+	G10965	G6535	chitinase-like protein	synonymous	n.d.

*n.d. not done

### Confirmation of variants

Similar to the confirmation of gene deletions, we analyzed virulent and attenuated *H. meleagridis* strains from monoxenic cultivation, which were used for NGS analysis, for the presence/absence of both truncating mutations, the SNP and the indel, by conventional PCR coupled with Sanger sequencing of PCR products (Table 2). In order to narrow the time point in which the mutations occurred, a set of xenic cultures of different passages spanning the period during which the attenuation occurred was used for this analysis. Both truncating mutations could be confirmed in the attenuated strain grown as monoxenic culture, whereas the gene was intact in the virulent strain grown under same conditions. Moreover, the analysis showed that both the SNP and the indel already occurred in xenic conditions with the indel appearing between passage 83 and 145, and the SNP occurring later between passages 145 and 237 (Fig. S2; Fig. S3). Similar to g6116_vir_ deletion, testing low and high passages of other unrelated *H. meleagridis* strains, grown as xenic clonal cultures, demonstrated that both the AGC Kinase and LRR loci did not change and remained as wild type throughout the in vitro cultivation (Table 2; Fig. S1).

### Characterization of protein classes

**Fig. 5:**
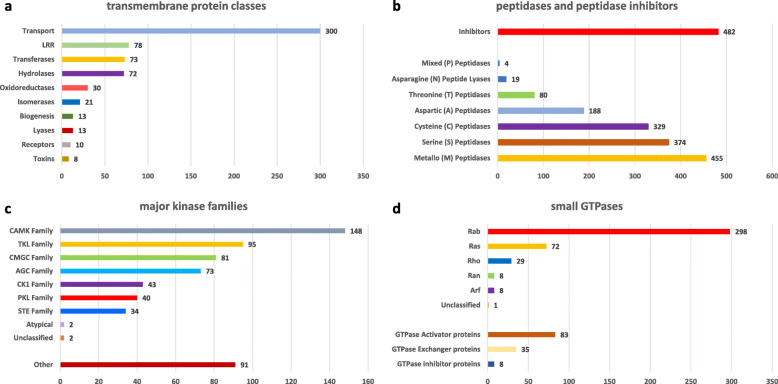
Classification of different protein classes in the *H. meleagridis* virulent strain: (**a**) ten most abundant categories of transmembrane proteins; (**b**) peptidases and peptidase inhibitors; (**c**) major kinase families; and (**d**) small GTPases

In addition to discovering differences between strains that might be responsible for attenuation, we exploited our new genomes to understand general features of *H. meleagridis* biology. As gene content was very similar between the two strains, we performed this and all following analyses considering only data from the virulent strain. Protein classes of specific relevance for parasitic protists include membrane proteins, peptidases, kinases and small GTPases, as they are often involved in processes necessary for colonization of the host, such as attachment and invasion [26]. Membrane proteins were determined by predicting transmembrane helices using TMHMM Server V2.0 and Phobius server, which identified 1331 and 1597 membrane proteins respectively, with majority of them (709 or 934) having only a single transmembrane helix (Table S2). The most abundant membrane proteins were membrane transporters (300 genes) followed by leucine-rich (LRR) domain-containing proteins (78 genes) (Fig. 5a). Since subfamily of LRR proteins, BspA-like proteins, are speculated to be involved in virulence of trichomonads [22] and are expanded in *T. vaginalis* [27], we hypothesized that among *H. meleagridis* membrane LRR proteins we identified some which might belong to that family. Thus, we looked for the presence of the BspA motif (LRR_5; PF13306) in these 78 genes with the online tool MOTIF (genome.jp/tools/motif/). This motif is a N-terminally located motif associated with BspA-like proteins. We found that 73/78 proteins contained the BspA motif, additionally we detected 4 membrane proteins not originally annotated as LRR proteins but containing the BspA motif (LRR_5), for a total of 77 BspA proteins containing at least one transmembrane domain. However, not all *H. meleagridis* genes encoding BspA-like proteins contained a transmembrane domain. A total of 94 BspA motif containing proteins were found in *H. meleagridis*, which leaves 17 proteins without predicted transmembrane helix. Next, we looked at peptidases, as they are involved in attachment to the host cell and invasion in *T. vaginalis* [28]. We detected 1449 peptidases classified into 7 different families, with metallo and serine peptidases being the most abundant (Fig. 5b). The analysis identified 482 peptidase inhibitors, with the majority of them (*n* = 293) being cysteine peptidase inhibitors. More than 50% of cysteine peptidase inhibitors belong to the Family I25B unassigned peptidase inhibitors that includes the cystatins of *T. vaginalis* (Table S3). Kinases are involved in cell signaling and cell cycle control and have been often proposed as drug targets in various parasitic protozoa [29]. We identified 609 kinases in *H. meleagridis*, which we further classified into functional classes (Fig. 5c; Table S4). Similar to other unicellular eukaryotes, the kinome repertoire of *H. meleagridis* includes most of the functional classes typical of eukaryotes but lacks proteins of the Tyrosine Kinase (TK) family. Finally, we found 416 small GTPases in *H. meleagridis*, with the vast majority belonging to the Rab subfamily (*n* = 298) (Fig. 5d; Table S5), which is consistent with a previous transcriptome study of *H. meleagridis* [8].

### Comparative genomics

**Fig. 6:**
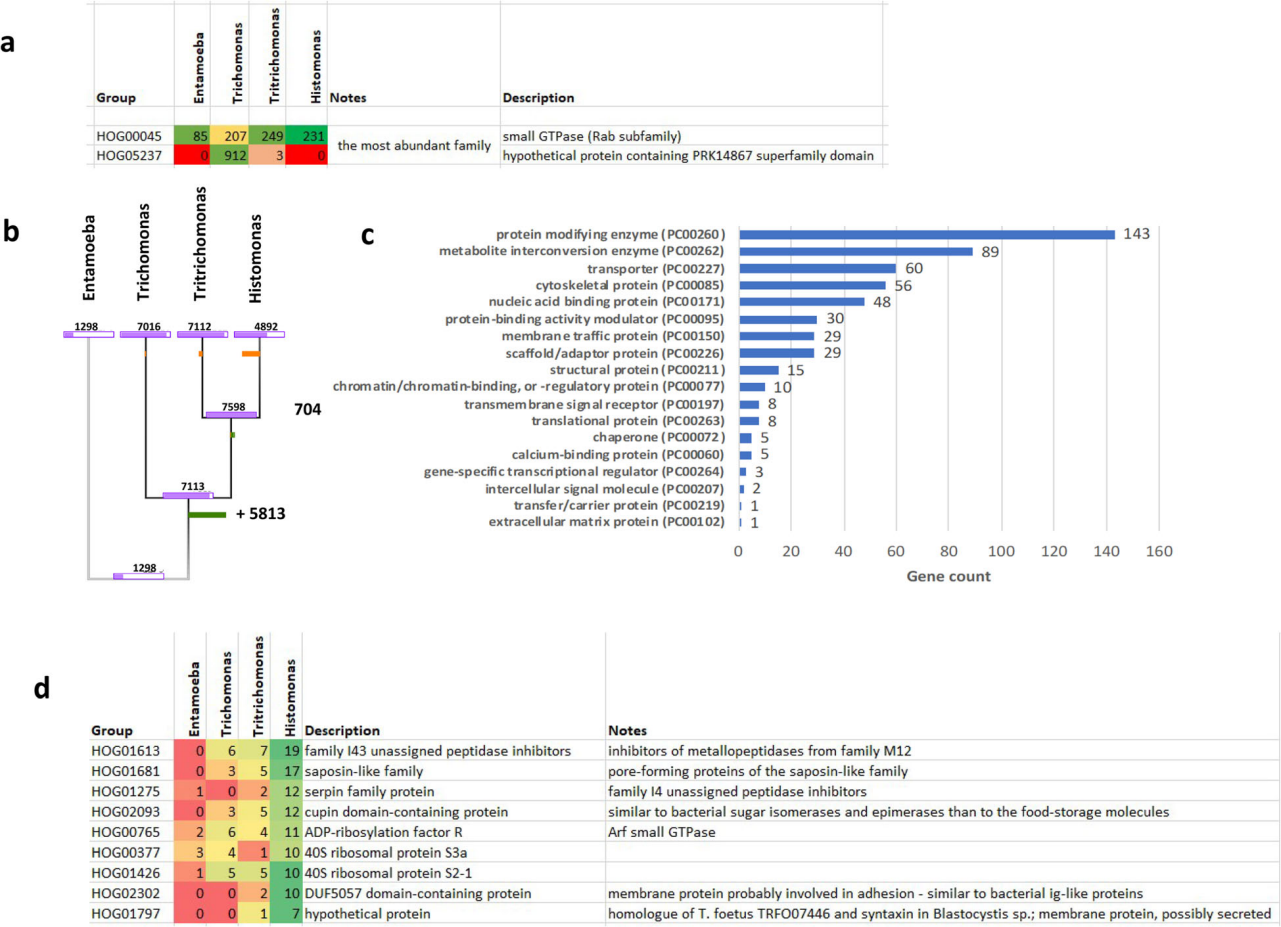
Comparative genomics of *H. meleagridis*: (**a**) most abundant gene families with gene family IDs (HOGs), number of genes in each species and description of the gene family; (**b**) phylogenetic tree displaying gains and losses where the number on the nodes/leaves show the total number of gene families present at that node/leaf; (**c**) classification of gene losses in different protein classes based on the *T. vaginalis* gene IDs (2627 orthologs) and (**d**) expanded gene families in *H. meleagridis* with gene family IDs (HOGs), number of genes in each species and description of expanded gene families in *H. meleagridis*;

The class Parabasalia includes parasitic and non-parasitic species. At the moment only two other genomes are completely sequenced: *Trichomonas vaginalis* and *Tritrichomonas foetus*. To get a global picture of gene family evolution in Parabasalia we clustered all protein sequences of *Histomonas meleagridis*, *Trichomonas vaginalis* and *Tritrichomonas foetus* including the outgroup *Entamoeba histolytica*, which is a unicellular parasite belonging to the phylum Amoebozoa. This analysis resulted in 7666 gene families, on which we performed a family gain/loss parsimony analysis to find lineage-specific losses and expansions. The most abundant protein/gene family in *H. meleagridis*, *T. foetus* and *E. histolytica* was Rab GTPase (HOG00045), with 231, 249 and 85 genes, respectively (Fig. 6a). In *T. vaginalis* the most abundant and also the most expanded gene family as compared to three other species is a hypothetical protein containing PRK14867 superfamily domain with 912 genes. Furthermore, we found 2704 gene families lost in the *H. meleagridis* branch, which is considerably higher compared to the lineage-specific losses in the two other Parabasalia species (Fig. 6b). To get an overview of the functions associated with these genes, we selected the gene IDs from the orthologs in *T. vaginalis* and performed a Gene List Analysis using the PANTHER online tool [30] (pantherdb.org/geneListAnalysis.do). The most abundant protein class were hydrolases (160 genes – 19.9%) (Fig. 6c) and contained cysteine proteases (29 genes) and metalloproteases (27 genes) as the most abundant subclasses. Additionally, we detected 8 expanded gene families in *H. meleagridis*, with at least 10 genes (Fig. 6d). These include family I43 unassigned peptidase inhibitors (HOG01613), pore-forming proteins of the saposin-like family (HOG01681), serpins serine and cysteine peptidase inhibitors of the family I4 (HOG01275), cupin domain-containing protein (HOG02093), Arf GTPase (HOG0765), 40S ribosomal protein S3a (HOG0377) and S2–1 (HOG01426) and a family of membrane proteins specific to *H. meleagridis* with similarity to bacterial Ig-like proteins (HOG02302) likely involved in adhesion.

## Discussion

The present study analysed the complete genome sequence of two closely related *H. meleagridis* strains, one displaying a virulent and the other an attenuated phenotype. The scope was to expand the genomic information on this poultry pathogen, which gained special attention after a substantial increase of outbreaks that resulted from the ban of effective chemotherapeutics. Considering the highly expanded nature of genomes of the only two parabasalids sequenced so far, *T. vaginalis* and *T. foetus*, we anticipated a similar feature for *H. meleagridis*. Therefore, we implemented the combination of ONT long reads and Illumina short reads in assembly of the genomes. Such approach enabled us to obtain megabase-sized contigs with high base-level accuracy with 100% of the genes having complete CDS. This clearly highlights the benefit of combining the long ONT reads with the short Illumina reads for the assembly, as protozoan genomes assembled only with Illumina reads still contain a substantial amount of partial CDS (i.e. [31]). Supporting this observation, the assembly of *H. meleagridis* genomes using only Illumina reads produced contigs of considerably shorter length (Illumina assembly longest contig = 113,223 bp vs Nanopore+Illumina longest contig = 3,384,422 bp for virulent strain; Table S6), underscoring the highest quality of the Nanopore+Illumina assembly. The genome size of *H. meleagridis* (43 Mb) is surprisingly small compared to its closely related species such as *T. vaginalis* (160 Mb) and *T. foetus* (161 Mb). A plausible hypothesis to explain this genome compactness is a much lower amount of genome duplication level in *H. meleagridis*, than in the other two parabasalid parasites. The advantage of having Mb-sized contigs is to compute directly the fraction of the genome that is duplicated instead of inferring this number by indirect methods (i.e. [14]). Indeed, the calculated overall genome duplication level in *H. meleagridis* of 20% for both completely sequenced strains is substantially lower than the reported duplication for *T. vaginalis* and *T. foetus*, with 65 and 61%, respectively [32].

The fact that both *H. meleagridis* strains stem from the same cell opened a venue to use the data for investigating genomic features of attenuation. The natural mechanisms of attenuation are poorly understood in eukaryotic pathogens, also due to the extreme complexity and diversity of unicellular eukaryotes as compared to bacteria and viruses. While a number of studies compared unrelated strains with different virulence profiles [33], only few addressed the specific scenario of attenuation of a single strain. For example, in *Babesia bovis*, a comparative transcriptomic study [34] of a virulent and a derived attenuated strain suggested that ves proteins, involved in cytoadherence to endothelial cells, could play a role in the generation of the attenuated phenotype. Changes in adherence proteins were also observed in *Trypanosoma cruzi* [35] where the attenuated strain displayed temporal asynchronicity in expression pattern of surface proteins such as trans-sialidases, mucins and mucin associated surface proteins. Transcriptional analysis of virulent and precocious (attenuated) strains of *Eimeria tenella* identified genes associated with cell survival, development or proliferation as strongly upregulated in the precocious strain [36]. A common feature of these studies is that they focused on gene expression changes, however the possibility of inheritable changes at the genomic level has been poorly investigated up to now.

Comparison of the two genomes with each other showed that the gene content is very similar between the two strains, which is not surprising considering that the origin of both strains can be traced to the same parent cell. But, it also singled out two coding regions that showed only partial support from Illumina reads in the attenuated strain. This indicated that during long term in vitro cultivation a deletion within these genes occurred in the virulent strain, which during this process became attenuated. Re-analysis of these loci confirmed the deletion in g6116_vir_ but not for g7085_vir_. Considering that g7085_vir_ as LRR domain-containing protein has several very similar orthologues in the genome we suspect that PCR-based re-analysis was hindered by the repetitive nature of LRR region and its existence on different loci in the genome. Thus, absence of involvement in the attenuation process cannot be ruled out for g7085_vir_. In addition to confirming the g6116_vir_ deletion in the attenuated strain we demonstrated that the deletion occurred already during in vitro cultivation in xenic conditions between passage 83 and 145, and not in the course of the monoxenization process through which both virulent and attenuated strains went independently [13]. The gene g6116_vir_ encodes for a hypothetical protein, however further BLAST searches revealed that the orthologue of g6116_vir_ in *T. vaginalis* encodes for a putative cell-surface adhesin. The probable membrane association of g6116_vir_ is supported by the presence of a transmembrane domain towards the C-terminus. This potential surface adhesin function and its loss in the attenuated strain would speak for its role in the virulence of the parasite, a hypothesis that needs to be tested in more detail. Aside from the possible role in virulence of *H. meleagridis*, the loss of protein encoded by g6116_vir_ might be supportive for the adaptation to the in vitro growth. Long-term cultivated parasites, which lack this gene, are much easier to cultivate and grow to much higher numbers in vitro than the parasites that are in culture for just few passages [37].

Further investigations on determining possible genes involved in the attenuation focused on the analysis of SNPs and indels between two genomes. A total of 13 homozygous nonsynonymous SNPs were detected in coding regions, with one of them resulting in the truncation of the protein in the attenuated strain due to the premature stop codon. The affected gene, g8794_vir /_ g337_att,_ encodes for LRR domain containing protein. As compared to its wild type partner in the virulent strain (g8794_vir_), the g337_att_ lacks a second LRR domain located at the C-terminus, and by that might represent a pseudogenization event. Since the transcript for this gene is detected in transcriptomes of both strains, it is possible that in the attenuated strain the transcript of g337_att_ might carry out a regulatory function or maybe even give rise to a protein of slightly different function. Considering that the prediction of functionality for this mutation by PROVEAN software tool was deleterious, the arise of the protein of slightly different function is dubious.

The analysis identifying indels that modify coding regions detected a single event, which due to the frameshift introduced an exchange of 38 amino acids and a premature stop codon. The gene g8786_vir_ and its counterpart in the attenuated strain (g346_att_), encode a serine/threonine kinase as judged by the presence of the protein kinase ATP binding site and the serine/threonine kinase site in both coding sequences. However, due to the indel the g346_att_ lacks a C-terminally located pleckstrin homology (PH) domain, which binds phosphoinositides and by that targets the protein to the cellular membrane. The frameshift that occurred in the mutated g346_att_ changed the last 38 amino acids, that now are predicted to form a short transmembrane domain at the C-terminus. Even though the attachment with the cell membrane is predicted for both, the mutant protein might assume a more fixed position as compared to the wild type which possibly changes its localization due to the transient interaction with phosphoinositides. The functionality prediction by PROVEAN tool characterized the mutant as deleterious, which is not entirely unexpected, since it lacks a complete domain. Generally, the combination of serine/threonine kinase catalytic domain at the N-terminus and the PH domain at the C-terminus is a hallmark of the phosphoinositide-dependent kinase 1 (PDK1), a kinase with a central role in cell-signaling [38]. In the case of the mutant, in which the PH domain is completely absent, the role as potential PDK1 would be compromised. Whether g8786_vir_ represents a true *H. meleagridis* homologue of this important regulatory molecule remains to be determined. The fact that its transcript could not be detected in the transcriptome of the in vitro grown parasites demonstrates a non-constitutive expression which is contradictory to PDK1, and suggests a potentially more specific role [8]. It is possible that g8786_vir_ /g346_att_ is only expressed in vivo, during the infection of the host. In such case its role in the virulence of the parasite can be hypothesized, which would be further supported by the predicted inefficacy of the mutant protein variant in the attenuated strain.

Other insightful results emerged from the analysis of proteins of different classes. The significance of kinases for a eukaryotic organism is reflected by the abundance of genes encoding for protein kinases. For *H. meleagridis* with more than 5% of the genome encoding kinases (609 genes), kinase relevance seems to be substantially high, when one considers 1.7% in human genome (518 genes) [39] or 1.5% in *T. vaginalis*, (880 kinases) [32] and 1.6% in *Plasmodium falciparum* (65 protein kinases) [40], highlighting the inherent complexity of this species. Aside to the *H. meleagridis* kinome, we also looked at the composition of membrane proteins, peptidases and small GTPases. In parasitic protozoa these proteins are often associated with processes relevant to colonization of the host, such as attachment and invasion. The majority of predicted membrane proteins in *H. meleagridis* contains only one transmembrane helix, a feature seen in *T. vaginalis* too [41]. Among predicted membrane proteins are the Leucine Rich Repeat (LRR) domain-containing proteins, which represent the second largest membrane protein family and potentially interesting in the aspect of parasite virulence. In *H. meleagridis*, all LRR proteins containing transmembrane helix are classified to BspA-like proteins, as they contain the specific BspA motif (LRR_5; PF13306) at the N-terminus. BspA are surface proteins of Bacteroidales/Spirochaetales involved in adhesion to the host tissue. They mediate host-pathogen interactions and cell aggregations. In trichomonads, precisely *T. vaginalis* and *Tetratrichomonas gallinarum* members of BspA-like proteins were shown to boost the attachment of parasites to host cells indicating a contribution to virulence [22]. In contrary to *T. vaginalis* and some other trichomonads in which only up to 30% of BspA-like proteins possess transmembrane helix [22], in *H. meleagridis* more than 80% of BspA-like proteins are predicted to be membrane-associated as they carry at least one transmembrane helix. This indicates their likely cell surface localization in *H. meleagridis,* which in turn advocates for the potential role in adhesion.

Genome data increased the catalogue of peptidases as previously identified from transcriptome, where only 115 peptidases were found [8]. Based on the search of the MEROPS database the number of peptidases in *H. meleagridis* seems quite high (*n* = 1449) as compared to *T. vaginalis* [32], which contains 446 peptidases. Such big discrepancy can be accounted to the fact that the current MEROPS database contains a much higher number of peptidases than at the time when the analyses of *T. vaginalis* genome sequence were performed. The MEROPS analysis also identified 482 putative peptidase inhibitors with the majority of them targeting cysteine peptidases, even though the biggest peptidase category for *H. meleagridis* are metallopeptidases. More than 50% of the cysteine peptidase inhibitors seem to be inhibitors of C1 and C13 peptidases, cathepsins and legumains. Considering that C1 and C13 peptidases represent only a minor portion of cysteine peptidases in *H. meleagridis*, this suggests a very tight control of *H. meleagridis* endogenous C1 and C13 peptidases. In respect to regulating peptidase activity in its environment, *H. meleagridis* might employ this high amount of specific cysteine peptidase inhibitors toward the legumains and cathepsins in the poultry caecum and by that protect itself from their activity.

In *H. meleagridis*, we found 416 small GTPases, with the vast majority belonging to the Rab subfamily (*n* = 298), consistent with a previous transcriptome study [8]. This number is comparable to *T. vaginalis*, which contains 356 small GTPases [32]. Furthermore, comparative genome analyses performed here identified the Rab GTPase (HOG00045) as the most abundant protein family in *H. meleagridis* (*n* = 231), *T. foetus* (*n* = 249) and *E. histolytica* (*n* = 85), underlining the complexity of membrane trafficking in these protozoan parasites. Even though it is not the most abundant protein family in *T. vaginalis*, the HOG00045 encoding Rab subfamily GTPases with 207 genes in *T. vaginalis* go in line with the highly intricate membrane trafficking system in this parasite too. Especially, when one compares it with about 70 members in higher eukaryotes such as humans [42].

When compared to *T. vaginalis*, *T. foetus* and *E. histolytica*, the genome of *H. meleagridis* lost 2704 gene families, which is more extensive as compared to the lineage-specific losses in the two other Parabasalia species (Fig. 6B). This correlates with the much smaller genome size and the relatively low level of genome duplication in *H. meleagridis*, as compared to *T. vaginalis* and *T. foetus*. The analysis furthermore demonstrated the complexity of parabasalids compared to the amoebozoan *E. histolytica*, since the complete group shows an expansion of 5813 gene families and the number of determined gene families for the amoeba is considerably smaller than for any of the three parabasalid species. Aside to lost gene families, the comparative genome analysis detected several gene families as expanded in *H. meleagridis*. A number of these gene families can be associated with a specific life cycle of the parasite, especially with its very close and intricate relationship with bacteria. *Histomonas meleagridis* requires the presence of bacteria for in vitro growth but also for colonizing the host tissues [43]. The relation between bacteria and *H. meleagridis* is multilateral, especially the role in nutrient supply is suggested to involve production of molecular byproducts that each partner uses for its survival. In that respect the expanded gene family of cupin domain-containing proteins could play a role, as these are more similar to bacterial sugar isomerases and epimerases than to the food-storage molecules. The other aspect, such as direct food source that bacteria represent for *H. meleagridis* could involve expanded gene families of pore-forming saposin–like proteins, Arf GTPase and bacterial Ig-like membrane proteins. Independent to the aspect of *Histomonas*/bacteria relation, some proteins within *H. meleagridis* lineage-specific expanded gene families might also constitute attractive drug target candidates in *H. meleagridis*.

## Conclusions

Combining the sequence data from two conceptually different sequencing platforms: Nanopore long reads and Illumina short reads enabled us to assemble high-quality genome sequences from two phenotypically different *H. meleagridis* strains, a parasite with limited sequence data available so far. Aside to the immense significance that the availability of the genome data represents for the research community focusing on *H. meleagridis*, here we present evidence for two gene deletions and two gene truncations differing between virulent and attenuated strain. These constitute attractive candidates for further experimental investigation to test the hypothesis that loss/change of function of these genes might have contributed to attenuation.

## Methods

### Protozoan cultures

All next generation sequencing experiments described in this paper were performed using virulent and attenuated, monoxenic mono-eukaryotic *H. meleagridis* cultures propagated in vitro, *H. meleagridis*/turkey/Austria/2922-C6/04-10x/18x-DH5α and *H. meleagridis*/turkey/Austria/2922-C6/04-290x/56x-DH5α, respectively [13]. The initial culture from which both, the virulent and the attenuated *H. meleagridis* strains, were obtained, was established in our laboratory in 2004 from faeces and caecal content of a backyard turkey suffering from histomonosis [12]. After few passages a single parasite cell was transferred via micromanipulation into a fresh medium containing caecal bacteria to obtain a xenic mono-eukaryotic clonal culture [12]. The virulent strain consists of parasites that were passaged for 10 passages as xenic mono-eukaryotic culture at which monoxenzation with *E. coli* DH5α was done and parasites were propagated in vitro for another 18 passages before harvesting for sequencing. The attenuated strain contains parasites that were passaged in vitro for 290 passages as xenic mono-eukaryotic culture after which monoxenization with *E. coli* DH5α was performed, afterwards parasites were in vitro cultivated for another 56 passages before harvesting for sequencing. For confirming the variants detected between the two strains, additional passages of *H. meleagridis*/turkey/Austria/2922-C6/04 together with 3 *H. meleagridis* strains listed in Table 4, all grown as xenic mono-eukaryotic cultures, were used. The cultures were incubated at 41 °C in Medium 199 containing Earl’s salts, (Gibco™, Invitrogen GmbH, Austria) supplemented with 15% heat-inactivated foetal bovine serum (FBS) (Gibco™, Invitrogen GmbH, Austria) and 0.25% of sterilized rice starch (Carl Roth GmbH + Co. KG, Germany). Cells were passaged every 3 days by transferring 2 ml of the old culture into a new T25 flask (Sarstedt, Inc., Germany).

**Table 4:** List of strains used in the present study. All listed strains are “mono-eukaryotic clonal cultures”, type of in vitro cultivation, xenic or monoxenic, is indicated. All *H. meleagridis* cultures were established in our laboratory from caecal material and faeces of infected turkeys or chickens that were sent to our Clinic (Clinic for Poultry and Fish Medicine, University of Veterinary Medicine, Vienna, Austria) for routine diagnostic investigation. The host bird species for each culture is indicated in the assignment of the strain according to following scheme: *Histomonas meleagridis* /bird species from which he parasite was isolated/country/diagnostic number – clone no. at micromanipulation/year of isolation

strain	type of in vitro cultivation	passage	purpose
*H. meleagridis*/turkey/Austria/2922-C6/04/DH5α^a^	monoxenic	28p	Illumina and MINIon sequencing, confirmation of variants
		346p	
*H. meleagridis*/turkey/Austria/2922-C6/04^b^	xenic	13p	confirmation of variants
		51p	
		83p	
		145p	
		237p	
		292p	
*H. meleagridis*/chicken/Austria13250-C20/10^c^	xenic	24p	
		316p	
*H. meleagridis*/turkey/Austria/2877-C3/05^c^	xenic	22p	
*H. meleagridis*/chicken/Austria/8175-C7/06^c^	xenic	21p	

^a^assignment: *Histomonas meleagridis* isolated from turkey/country/diagnostic number – clone no. at micromanipulation/year of isolation/monoxenic grown with *E. coli* DH5α. Monoxenization was done independently at passage 10 (virulent) and 290 (attenuated) [13]

^b^xenic cultures prior to monoxenization at passage 290

^c^ assignment *Histomonas meleagridis* /bird species from which he parasite was isolated/country/diagnostic number – clone no. at micromanipulation/year of isolation

For Illumina and MINion sequencing, monoxenic virulent and attenuated *H. meleagridis* were harvested at passage number 28 (28p) and 346 (346p), respectively. In order to remove majority of the bacteria, *Histomonas* cells were purified through a series of washing steps using pre-warmed M199 media without serum [9]. The cell pellets were immediately frozen at − 80 °C until further use.

### DNA extraction

For Illumina sequencing, DNA was extracted with QIAamp DNA Mini Kit (Qiagen, Hilden, Germany) according to manufacturer’s recommendation. The extraction of high molecular weight DNA, used for MINion sequencing, was performed according to a previously published protocol implementing slight modifications [44]. Briefly, 10^8^ cells were pelleted by centrifugation at 4500×g for 10 min. The cells were re-suspended in 10 ml TLB (10 mM Tris-Cl pH 8.0, 25 mM EDTA pH 8.0, 0.5% (w/v) SDS, 20 μg/ml RNase A (Qiagen, Hilden, Germany)), vortexed at full speed for 5 s and incubated at 37 °C for 1 h, with gentle mix by inversion every 30 min. Then, complete 100 μl of Proteinase K (Qiagen, Hilden, Germany) were added to obtain a final concentration of 200 μg/ml. The solution was gently mixed by inversion and incubated at 50 °C for 2 h, with slow end-over-end rotations every 30 min. After completion, the sample was centrifuged at 200×g, and the viscous supernatant containing cell lysate was distributed into two 15 ml Falcon tubes prepared with phase-lock gel. Then, 5 ml of TE-saturated phenol pH 7.5 (Sigma-Aldrich) were added to the lysates and placed on a rotator at 20 rpm for 10 min. The preparations were centrifuged at 3000×g for 10 min and the aqueous phases were poured into two new 15 ml tubes containing phase-lock gel; followed by the addition of 2.5 ml buffer saturated phenol pH 7.5 and 2.5 ml chloroform-isoamyl alcohol 24:1 mix to each tube. Phase separation was carried out as described above and both aqueous phases were combined. The DNA was precipitated by the addition of 4 ml 5 M ammonium acetate and 30 ml ice-cold 96% ethanol, for 4 days at − 20 °C. Precipitated DNA was collected by centrifugation at 10,000×g and washed twice in 70% ethanol. After the final spin down, the sample was air dried for 15 min at room temperature and 200 μl EB (10 mM Tris-Cl pH 8, 0.02% (v/v) Triton X-100) were added to the DNA pellet that was kept at + 4 °C until completely dissolved. DNA quantity and quality were assessed using Qubit™ dsDNA BR Assay Kit (Invitrogen, Life technologies), NanoDrop 2000 (Thermo Fisher Scientific) and Agilent 4200 TapeStation System using Genomic DNA Screen tape (Agilent technologies). The extraction of DNA for verifying the variants was performed by DNeasy Blood and Tissue Kit (Qiagen, Hilden, Germany) according to manufacturer’s instructions.

### Illumina sequencing

Illumina library preparation and sequencing were performed at Biomedical Sequencing Facility (Vienna, Austria). For each *H. meleagridis* strain a paired-end Illumina library was prepared from 1.5 μg DNA using TruSeq DNA PCR-Free Library Kit (Illumina) according to manufacturer’s recommendations. Sequencing (150 bp PE) was carried out on Illumina HiSeq 3000/4000 platform. Resulting reads were imported into CLC Genomics Workbench 12.0 (https://www.qiagenbioinformatics.com/), quality trimmed and adapters were removed. The processed reads were assembled into contigs using the De Novo Assembly workflow (default parameters).

### Nanopore sequencing

Libraries for Nanopore sequencing were prepared from 0.8 μg of high molecular weight *H. meleagridis* DNA using SQK-LSK109 1D ligation kit (Oxford Nanopore Technologies, Oxford, UK) according to manufacturer’s recommendation. Libraries were sheared by using the g-TUBE (Covaris) and centrifuged at 6000 rpm in an Eppendorf 5424 centrifuge for 2 × 1 min, inverting the tube between centrifugations. DNA repair (NEBNext® FFPE DNA Repair Mix, M6630S, New England Biolabs GmbH, Frankfurt am Main, Germany) and End repair / dA-tailing (NEBNext® Ultra™ II End Repair/dA-Tailing Module, New England Biolabs GmbH, Frankfurt am Main, Germany) were performed by adding 27 μl nuclease-free water (NFW), 3.5 μl FFPE Repair Buffer, 2 μl FFPE DNA Repair Mix, 3.5 μl Ultra II End-prep reaction buffer and 3 μl Ultra II End-prep enzyme mix to 20ul of the previously sheared DNA in a 0.2 ml thin-walled PCR tube. Using a thermocycler, the mixture was incubated at 20 °C for 5 min and 65 °C for 5 min. The preparation was transferred to a 1.5 ml Eppendorf DNA LoBind tube and cleaned up using a 60 μl of Agencourt AMPure XP beads (Beckman Coulter Life Sciences, Vienna, Austria), incubated at room temperature with end over end mixing for 5 min, washed twice with 200 μl fresh 70% ethanol and allowed to air dry for 30 s. Adapter Ligation was performed by adding 60 μl DNA sample from the previous step, 25 μl Ligation Buffer (LNB), 10 μl T4 DNA Ligase (NEBNext® Quick Ligation Module) and 5 μl Adapter Mix (AMX) in a 1.5 ml Eppendorf DNA LoBind tube, incubating the preparation at room temperature for 10 min. The adaptor-ligated DNA was cleaned up by adding 40 μl of Agencourt AMPure XP beads (Beckman Coulter, Life Sciences, Vienna, Austria), incubated at room temperature with end over end mixing for 5 min. The beads were washed twice with 250 μl Long Fragment Buffer (LFB) and allowed to air dry for 30 s. The DNA was eluted by adding 15 μl Elution Buffer (EB) and incubated for 10 min at 37 °C. Flow Cell Priming was carried out with the introduction of 800 μl of the priming mix into the flow cell via the priming port, with a 5-min incubation period. The DNA Library was prepared for loading adding 37.5 μl Sequencing Buffer (SQB) and 25.5 μl Loading Beads (LB) to 12 μl of the previously generated DNA Library. Flow cells (FLO-MIN106) were run with the standard MinKNOW software. Base calling option was enabled for a run duration of ∼48 h, with a Bias Voltage of -180 mV and time between mux scans of 1.5 h.

### Genome assembly and annotation

For processing of Nanopore reads, FAST5 files were converted to FASTQ using the Guppy basecaller followed by adapters trimming with Porechop (https://github.com/rrwick/Porechop). A draft assembly was generated using Flye (parameters -g 50 m --meta) from the Nanopore reads. To scan for *E. coli* contigs and contaminations, the draft assembly was divided into 1Kb windows and analyzed through the Taxonomic Profiling tool of CLC Genomics Workbench 12, Microbial Genomics Module (Qiagen, Hilden, Germany) against the complete bacterial genome database. Contigs matching in their entirety to bacteria were removed from the draft assembly. For assembly refining, Illumina reads were subsampled to 30 million and aligned to the draft assembly by minimap2 [45] (default parameters) followed by three rounds of refining with racon [46] (default parameters). The quality of the refined assembly was evaluated in the following way: a reference assembly made only from Illumina reads was constructed using the De Novo Assembly tool (default parameters) from CLC Genomics Workbench 12 (Qiagen, Hilden, Germany) (https://www.qiagenbioinformatics.com/), as Illumina reads have higher base-level accuracy compared to Nanopore reads. Then, the average similarity between the refined assembly and the Illumina assembly was computed by the program dnadiff of the MUMmer package [15]. To evaluate genome completeness, a set of eukaryotic core genes was downloaded from the CEGMA web page (http://korflab.ucdavis.edu/datasets/cegma/core/core.fa) and BLASTed to the refined assembly (E-value < 10^− 3^) in order to estimate the percentage of eukaryotic core genes in the refined assembly. For genome annotation, the transcriptome dataset from Mazumdar et al. [8] was used as input for training of the AUGUSTUS gene predictor using the web interface of this tool [47]. After training, the local version of AUGUSTUS [48] was run on the refined assembly of each strain (parameters --strand = both --genemodel = partial). Functional annotation of coding genes was made using the Blast2GO tool from CLC Genomics Workbench 12 (Qiagen, Germany). Annotation of repetitive sequences and transposons was performed by RepeatMasker 4.0.7 assessed in November 2019 (http://www.repeatmasker.org/) (parameters --species “trichomonas”) [49].

### Identification of variants between strains

For variants identification (SNPs and indels), the assembly of the attenuated strain was aligned against the virulent strain using nucmer from the MUMmer package [15] followed by variant calling with the show-snps tool (parameters -C -l -r). To validate the accuracy of variant calling, Illumina reads from both strains were aligned to the assembly of the virulent strain with Bowtie2 [50] (default parameters). Then, variants with minimum coverage of 30 in both strains and minimum reference allele frequency of 80% were regarded as true homozygous SNPs (or indels) between the two strains. SNPs located in coding regions were extracted and their potential impact on protein stability was predicted using the online tool PROVEAN [25].

### Confirmation of variants between strains

In order to validate variants (1 indel, 1 SNP) that caused premature stops in the corresponding coding regions, conventional PCRs were performed. To confirm the two deletions, conventional and real-time PCRs using primers and probes listed in Table S7 were employed. Real-time PCRs were done in 20 μl reaction mixture on the AriaMx real time cycler (Agilent Technologies, USA) using Brilliant III UltraFast qPCR Master Mix (Agilent Technologies, USA) with 30 nM ROX as reference dye, 500 nM primers and 100 nM TaqMan probe (Table S7). Thermal profile of real-time reactions was as follows: 15-min at 95 °C, followed by 40 cycles of 15 s at 95 °C and of 30 s at 60 °C. Fluorescent signal was detected at each cycle during the 60 °C step. All conventional PCRs were performed in 25 μl reaction by using HotStar Taq Master Mix Kit (Qiagen, Vienna, Austria) and 0.4 μM of each primer (Table S7). Thermo-cycling conditions for all conventional PCRs were: one cycle of 95 °C for 15 min; 40 cycles of 95 °C for 30 s, 51 °C or 52 °C (depending on the target region, Table S7) for 30 s and 72 °C for 1 min; followed by final elongation step at 72 °C for 10 min. Amplification products (25 μl) were electrophoresed in a 1.0% Tris acetate-EDTA-agarose gel, stained with ethidium bromide and visualized under UV light (Biorad Universal Hood II, Bio-Rad Laboratories, California, USA). Fragment sizes were determined with reference to a 1 kb ladder (Invitrogen, Life Technologies, Austria). Amplification products (25 μl) were electrophoresed in a 1.0% Tris acetate-EDTA-agarose gel, stained with ethidium bromide and visualized under UV light (Biorad Universal Hood II, Bio-Rad Laboratories, California, USA). Fragment sizes were determined with reference to a 1 kb ladder (Invitrogen, Life Technologies, Austria). Amplicons of the expected sizes were cut from the gel and extracted using the QIAquick Gel Extraction Kit (Qiagen, Vienna, Austria) according to the manufacturer’s instructions. Sanger sequencing was performed by LGC Genomics GmbH (Berlin, Germany) employing the PCR primers.

### Characterization of protein classes

Since gene content between the virulent and the attenuated *H. meleagridis* strain was very similar, we performed all following analyses for characterizing different protein classes using only data from the virulent strain. Transmembrane proteins were detected by predicting transmembrane helices employing the TMHMM web server v2.0 (http://www.cbs.dtu.dk/services/TMHMM/) and the Phobius web server, a combined transmembrane topology and signal peptide predictor (http://phobius.sbc.su.se/) [51]. In order to identify BspA-like proteins, we looked for the presence of BspA motifs in all LRR proteins with the online tool MOTIF (genome.jp/tools/motif/). Kinases were extracted from the functional annotation by selecting proteins containing the IPR000719 (kinase) or IPR011009 (kinase-like) domains. Additionally, the *H. meleagridis* proteome was compared to the full *T. vaginalis* kinase proteins database (KinBase The Kinase Database (retrieved 2020, July 24) http://kinase.com/web/current/kinbase), with BLASTP (E-Value-Hit-Filter of ≤10^− 10^). Hits showing ≥50% similarity , were added to the list containing sequences with the IPR000719 (kinase) or IPR011009 (kinase-like) domains. Results were further classified into families according to the classification of the Wikinome web page (http://kinase.com/wiki/index.php/Kinase_classification). Small GTPases were identified by screening all protein sequences with the tool GTPasePred [52]. The classification into major classes was based on the sequence description provided by Blast2gGO and the InterProScan codes attributed to each sequence. To identify peptidases, all *H. meleagridis* proteins were compared to the MEROPS “pepunit.lib” database (MEROPS the Peptidase Database (retrieved 2020, July 20) https://www.ebi.ac.uk/merops/download_list.shtml) [53] with BLASTP (E-value < 10^− 10^) and hits with at least 50% similarity to the reference peptidase were extracted.

### Comparative genomics

Protein sequences from *E. histolytica* and *T. vaginalis* were downloaded from AmoebaDB [54] (version 46) and TrichDB [55] (version 46), respectively. Protein sequences for *T. foetus* were downloaded from https://www.labinfo.lncc.br/tritrichomonas_foetus/. For all species, proteins corresponding to the longest transcripts for each gene were extracted and pseudogenes were removed. The tool OMA [19] was run on the protein datasets of *E. histolytica*, *T. vaginalis*, *T. foetus* and *H. meleagridis* in order to compute gene families. For *H. meleagridis*, only the proteins of the virulent strain were employed. A Dollo parsimony approach was applied to the gene family data to estimate gain/losses along the species tree using the program Count [56]. Functional enrichment for subsets of gene family gains/losses was analyzed with the online tool PANTHER (http://www.pantherdb.org/) using *T. vaginalis* gene IDs as a reference [30].

## Supplementary Information

**Additional file 1: Fig. S1.** Confirmation of deletions by PCR. Agarose gel electrophoresis of PCR for (A) g6116_vir_ and (B) g7085_vir_ loci.

**Additional file 2: Fig. S2.** Nucleotide alignment of the SNP region.

**Additional file 3: Fig. S3.** Nucleotide alignment of the indel region.

**Additional file 4: Table S1.** Annotation of transposons in the virulent and attenuated strains of *H. meleagridis.*

**Additional file 5: Table S2.** Transmembrane proteins in *H. meleagridis.*

**Additional file 6: Table S3.** Peptidases in *H. meleagridis.*

**Additional file 7: Table S4.** Kinases in *H. meleagridis.*

**Additional file 8: Table S5.** GTPases in *H. meleagridis.*

**Additional file 9: Table S6.** Statistics of genome assemblies for virulent and attenuated strain using only Illumina reads.

**Additional file 10: Table S7.** List of primers and probes.

## Data Availability

The Whole Genome Shotgun project (BioProject PRJNA594289, BioSamples SAMN13511252 and SAMN135111566) has been deposited at DDBJ/ENA/GenBank under following accessions WSYJ00000000 and JAANOX000000000. Raw reads are available in Short Reads Archive under following accession numbers: SRX12366314- SRX12366315; SRX12373195- SRX12373196.

## Abbreviations

SNP: Single nucleotide polymorphism
ONT: Oxford Nanpore Technology
Mb: Megabase
LRR: Leucine rich repeat
CEGMA: Core Eukaryotic Genes Mapping Approach
BUSCO: Benchmarking Universal Single-Copy Orthologs
CDS: Coding sequence
PCR: Polymerase chain reaction
ATP: Adenosine-5′-triphosphate
GTP: Guanosine-5′-triphosphate
BLAST: Basic Local Alignment Search Tool
TK: Tyrosine kinase
PH: Pleckstrin homology
PDK1: Phosphoinositide dependent kinase 1
EDTA: Ethylenediaminetetraacetic acid
UV: Ultraviolet
kb: kilobase
μl: microliter
