# Supplementary figures and images for: Complete genomes of the eukaryotic poultry parasite Histomonas meleagridis: linking sequence analysis with virulence / attenuation
Source: BMC Genomics. 2021 Oct 21;22:753. doi: 10.1186/s12864-021-08059-2 (PMC8529796; doi:10.1186/s12864-021-08059-2)

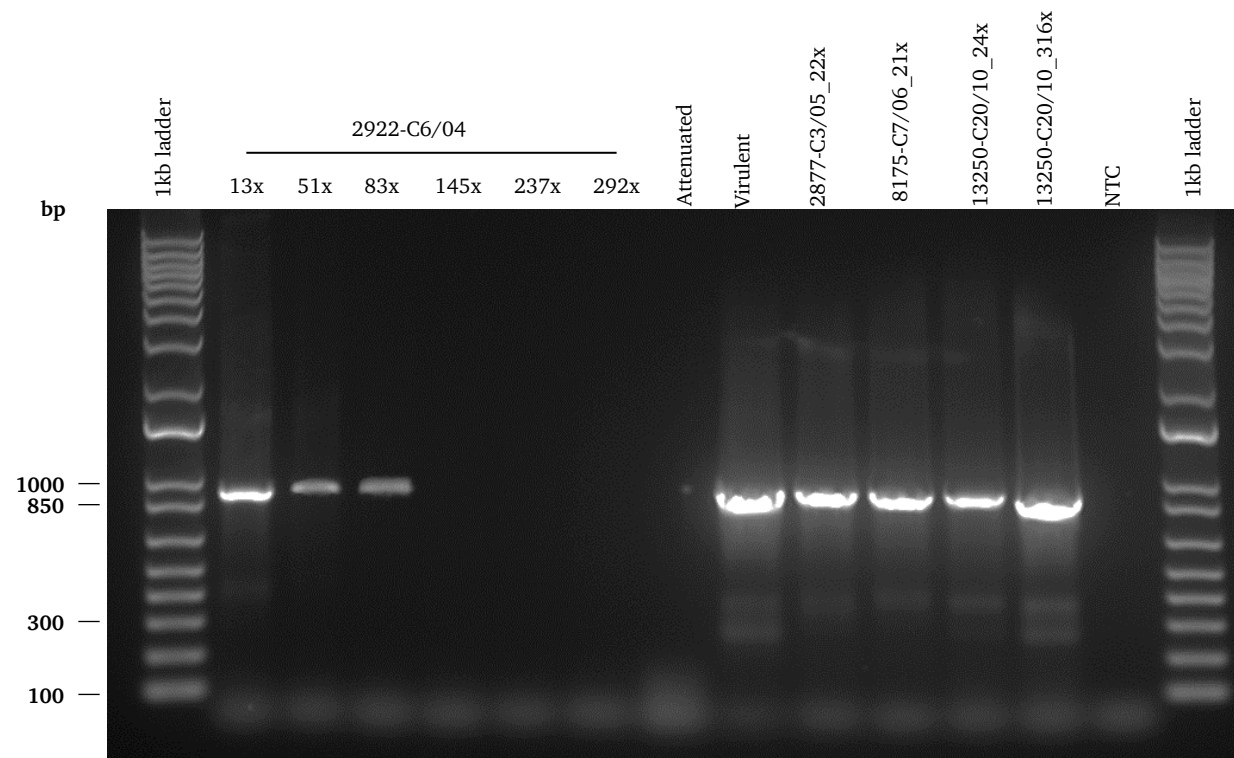

Supplement: Supplementary file 1 — Additional file 1: Fig. S1. Confirmation of deletions by PCR. Agarose gel electrophoresis of PCR for (A) g6116vir and (B) g7085vir loci. [file 12864_2021_8059_MOESM1_ESM.pdf]
