# Supplementary material for: Complete genomes of the eukaryotic poultry parasite Histomonas meleagridis: linking sequence analysis with virulence / attenuation
Source: BMC Genomics. 2021 Oct 21;22:753. doi: 10.1186/s12864-021-08059-2 (PMC8529796; doi:10.1186/s12864-021-08059-2)
Supplement: Supplementary file 3 — Additional file 3: Fig. S3. Nucleotide alignment of the indel region. [file 12864_2021_8059_MOESM3_ESM.pdf]

Sequence	1–120	
Consensus	CGTTCTTCTCTCAGATTTTGGTACAGCCCTAATTGAAAAATCTGATAGTCAAGAATTAAATCGTTCATCCATTGTTGGAACTCCAGCCTTTGTTGCCCCAGAGCTCCTTAATGATGGAAA	
	10 20 30 40 50 60 70 80 90 100 110 120	
virulent_2922-C6/04/DH5alpha_24x	CGTTCTTCTCTCAGATTTTGGTACAGCCCTAATTGAAAAATCTGATAGTCAAGAATTAAATCGTTCATCCATTGTTGGAACTCCAGCCTTTGTTGCCCCAGAGCTCCTTAATGATGGAAA	120
attenuated_2922-C6/04/DH5alpha_295x	CGTTCTTCTCTCAGATTTTGGTACAGCCCTAATTGAAAAATCTGATAGTCAAGAATTAAATCGTTCATCCATTGTTG-AACTCCAGCCTTTGTTGCCCCAGAGCTCCTTAATGATGGAAA	119
2922-C6/04_13x	CGTTCTTCTCTCAGATTTTGGTACAGCCCTAATTGAAAAATCTGATAGTCAAGAATTAAATCGTTCATCCATTGTTGGAACTCCAGCCTTTGTTGCCCCAGAGCTCCTTAATGATGGAAA	120
2922-C6/04_51x	CGTTCTTCTCTCAGATTTTGGTACAGCCCTAATTGAAAAATCTGATAGTCAAGAATTAAATCGTTCATCCATTGTTGGAACTCCAGCCTTTGTTGCCCCAGAGCTCCTTAATGATGGAAA	120
2922-C6/04_83x	CGTTCTTCTCTCAGATTTTGGTACAGCCCTAATTGAAAAATCTGATAGTCAAGAATTAAATCGTTCATCCATTGTTGGAACTCCAGCCTTTGTTGCCCCAGAGCTCCTTAATGATGGAAA	120
2922-C6/04_145x	CGTTCTTCTCTCAGATTTTGGTACAGCCCTAATTGAAAAATCTGATAGTCAAGAATTAAATCGTTCATCCATTGTTG-AACTCCAGCCTTTGTTGCCCCAGAGCTCCTTAATGATGGAAA	119
2922-C6/04_237x	CGTTCTTCTCTCAGATTTTGGTACAGCCCTAATTGAAAAATCTGATAGTCAAGAATTAAATCGTTCATCCATTGTTG-AACTCCAGCCTTTGTTGCCCCAGAGCTCCTTAATGATGGAAA	119
2922-C6/04_292x	CGTTCTTCTCTCAGATTTTGGTACAGCCCTAATTGAAAAATCTGATAGTCAAGAATTAAATCGTTCATCCATTGTTG-AACTCCAGCCTTTGTTGCCCCAGAGCTCCTTAATGATGGAAA	119
8175-C7/06_21x	CGTTCTTCTCTCAGATTTTGGTACAGCCCTAATTGAAAAATCTGATAGTCAAGAATTAAATCGTTCATCCATTGTTGGAACTCCAGCCTTTGTTGCCCCAGAGCTCCTTAATGATGGAAA	120
2877-C3/05_24x	CGTTCTTCTCTCAGATTTTGGTACAGCCCTAATTGAAAAATCTGATAGTCAAGAATTAAATCGTTCATCCATTGTTGGAACTCCAGCCTTTGTTGCCCCAGAGCTCCTTAATGATGGAAA	120
13250-C20/10_24x	CGTTCTTCTCTCAGATTTTGGTACAGCCCTAATTGAAAAATCTGATAGTCAAGAATTAAATCGTTCATCCATTGTTGGAACTCCAGCCTTTGTTGCCCCAGAGCTCCTTAATGATGGAAA	120
13250-C20/10_316x	CGTTCTTCTCTCAGATTTTGGTACAGCCCTAATTGAAAAATCTGATAGTCAAGAATTAAATCGTTCATCCATTGTTGGAACTCCAGCCTTTGTTGCCCCAGAGCTCCTTAATGATGGAAA	120

Sequence	121–240	
Consensus	AATCTGTTATAGTTCTGATATGTGGTCATTTGGTTGTGTCATATTCAATCTTCTTACAGGCACAGCACCTTTTAGTGGACAAAACACAGTTGAATTAATGAACAACATCACTGAGCTTAA	
	130 140 150 160 170 180 190 200 210 220 230 240	
virulent_2922-C6/04/DH5alpha_24x	AATCTGTTATAGTTCTGATATGTGGTCATTTGGTTGTGTCATATTCAATCTTCTTACAGGCACAGCACCTTTTAGTGGACAAAACACAGTTGAATTAATGAACAACATCACTGAGCTTAA	240
attenuated_2922-C6/04/DH5alpha_295x	AATCTGTTATAGTTCTGATATGTGGTCATTTGGTTGTGTCATATTCAATCTTCTTACAGGCACAGCACCTTTTAGTGGACAAAACACAGTTGAATTAATGAACAACATCACTGAGCTTAA	239
2922-C6/04_13x	AATCTGTTATAGTTCTGATATGTGGTCATTTGGTTGTGTCATATTCAATCTTCTTACAGGCACAGCACCTTTTAGTGGACAAAACACAGTTGAATTAATGAACAACATCACTGAGCTTAA	240
2922-C6/04_51x	AATCTGTTATAGTTCTGATATGTGGTCATTTGGTTGTGTCATATTCAATCTTCTTACAGGCACAGCACCTTTTAGTGGACAAAACACAGTTGAATTAATGAACAACATCACTGAGCTTAA	240
2922-C6/04_83x	AATCTGTTATAGTTCTGATATGTGGTCATTTGGTTGTGTCATATTCAATCTTCTTACAGGCACAGCACCTTTTAGTGGACAAAACACAGTTGAATTAATGAACAACATCACTGAGCTTAA	240
2922-C6/04_145x	AATCTGTTATAGTTCTGATATGTGGTCATTTGGTTGTGTCATATTCAATCTTCTTACAGGCACAGCACCTTTTAGTGGACAAAACACAGTTGAATTAATGAACAACATCACTGAGCTTAA	239
2922-C6/04_237x	AATCTGTTATAGTTCTGATATGTGGTCATTTGGTTGTGTCATATTCAATCTTCTTACAGGCACAGCACCTTTTAGTGGACAAAACACAGTTGAATTAATGAACAACATCACTGAGCTTAA	239
2922-C6/04_292x	AATCTGTTATAGTTCTGATATGTGGTCATTTGGTTGTGTCATATTCAATCTTCTTACAGGCACAGCACCTTTTAGTGGACAAAACACAGTTGAATTAATGAACAACATCACTGAGCTTAA	239
8175-C7/06_21x	AATCTGTTATAGTTCTGATATGTGGTCATTTGGTTGTGTCATATTCAATCTTCTTACAGGCACAGCACCTTTTAGTGGACAAAACACAGTTGAATTAATGAACAACATCACTGAGCTTAA	240
2877-C3/05_24x	AATCTGTTATAGTTCTGATATGTGGTCATTTGGTTGTGTCATATTCAATCTTCTTACAGGCACAGCACCTTTTAGTGGACAAAACACAGTTGAATTAATGAACAACATCACTGAGCTTAA	240
13250-C20/10_24x	AATCTGTTATAGTTCTGATATGTGGTCATTTGGTTGTGTCATATTCAATCTTCTTACAGGCACAGCACCTTTTAGTGGACAAAACACAGTTGAATTAATGAACAACATCACTGAGCTTAA	240
13250-C20/10_316x	AATCTGTTATAGTTCTGATATGTGGTCATTTGGTTGTGTCATATTCAATCTTCTTACAGGCACAGCACCTTTTAGTGGACAAAACACAGTTGAATTAATGAACAACATCACTGAGCTTAA	240

Sequence	241–344	
Consensus	GTTTAATCCAGTCATTAAAACACTTCCGAAAACCGCCAAAGATCTCATCACATCCTTACTTAAGCTTGATCCACACGAGCGTATTGGATACGGGGAAGCCAAAG	
	250 260 270 280 290 300 310 320 330 340	
virulent_2922-C6/04/DH5alpha_24x	GTTTAATCCAGTCATTAAAACACTTCCGAAAACCGCCAAAGATCTCATCACATCCTTACTTAAGCTTGATCCACACGAGCGTATTGGATACGGGGAAGCCAAAG	344
attenuated_2922-C6/04/DH5alpha_295x	GTTTAATCCAGTCATTAAAACACTTCCGAAAACCGCCAAAGATCTCATCACATCCTTACTTAAGCTTGATCCACACGAGCGTATTGGATACGGGGAAGCCAAAG	343
2922-C6/04_13x	GTTTAATCCAGTCATTAAAACACTTCCGAAAACCGCCAAAGATCTCATCACATCCTTACTTAAGCTTGATCCACACGAGCGTATTGGATACGGGGAAGCCAAAG	344
2922-C6/04_51x	GTTTAATCCAGTCATTAAAACACTTCCGAAAACCGCCAAAGATCTCATCACATCCTTACTTAAGCTTGATCCACACGAGCGTATTGGATACGGGGAAGCCAAAG	344
2922-C6/04_83x	GTTTAATCCAGTCATTAAAACACTTCCGAAAACCGCCAAAGATCTCATCACATCCTTACTTAAGCTTGATCCACACGAGCGTATTGGATACGGGGAAGCCAAAG	344
2922-C6/04_145x	GTTTAATCCAGTCATTAAAACACTTCCGAAAACCGCCAAAGATCTCATCACATCCTTACTTAAGCTTGATCCACACGAGCGTATTGGATACGGGGAAGCCAAAG	343
2922-C6/04_237x	GTTTAATCCAGTCATTAAAACACTTCCGAAAACCGCCAAAGATCTCATCACATCCTTACTTAAGCTTGATCCACACGAGCGTATTGGATACGGGGAAGCCAAAG	343
2922-C6/04_292x	GTTTAATCCAGTCATTAAAACACTTCCGAAAACCGCCAAAGATCTCATCACATCCTTACTTAAGCTTGATCCACACGAGCGTATTGGATACGGGGAAGCCAAAG	343
8175-C7/06_21x	GTTTAATCCAGTCATTAAAACACTTCCGAAAACCGCCAAAGATCTCATCACATCCTTACTTAAGCTTGATCCACACGAGCGTATTGGATACGGGGAAGCCAAAG	344
2877-C3/05_24x	GTTTAATCCAGTCATTAAAACACTTCCGAAAACCGCCAAAGATCTCATCACATCCTTACTTAAGCTTGATCCACACGAGCGTATTGGATACGGGGAAGCCAAAG	344
13250-C20/10_24x	GTTTAATCCAGTCATTAAAACACTTCCGAAAACCGCCAAAGATCTCATCACATCCTTACTTAAGCTTGATCCACACGAGCGTATTGGATACGGGGAAGCCAAAG	344
13250-C20/10_316x	GTTTAATCCAGTCATTAAAACACTTCCGAAAACCGCCAAAGATCTCATCACATCCTTACTTAAGCTTGATCCACACGAGCGTATTGGATACGGGGAAGCCAAAG	344
