# Supplementary material for: Complete genomes of the eukaryotic poultry parasite Histomonas meleagridis: linking sequence analysis with virulence / attenuation
Source: BMC Genomics. 2021 Oct 21;22:753. doi: 10.1186/s12864-021-08059-2 (PMC8529796; doi:10.1186/s12864-021-08059-2)
Supplement: Supplementary file 9 — Additional file 9: Table S6. Statistics of genome assemblies for virulent and attenuated strain using only Illumina reads. [file 12864_2021_8059_MOESM9_ESM.docx]

**S6 Table** Statistics of genome assemblies for virulent and attenuated strain using only Illumina reads

| **genomic feature** | **virulent strain** | **attenuated strain** |
| --- | --- | --- |
| number of contigs | 3,445 | 3,456 |
| largest contig (bp) | 131,223 | 182,599 |
| total length (bp) | 30,269,295 | 40,256,341 |
| N50 (bp) | 31,733 | 31,203 |
| GC content | 28.91 | 28.93 |
| number of genes | 11,848 | 11,821 |
| number of exons | 13,670 | 13,655 |
| number of introns | 1,942 | 1,955 |
| number of CDS | 13,670 | 13,655 |
| start codons | 11,632 | 11,602 |
| stop codons | 11,571 | 11,821 |
