# Supplementary material for: Complete genomes of the eukaryotic poultry parasite Histomonas meleagridis: linking sequence analysis with virulence / attenuation
Source: BMC Genomics. 2021 Oct 21;22:753. doi: 10.1186/s12864-021-08059-2 (PMC8529796; doi:10.1186/s12864-021-08059-2)
Supplement: Supplementary file 10 — Additional file 10: Table S7. List of primers and probes. [file 12864_2021_8059_MOESM10_ESM.docx]

Table S7 Primers and probes used in the present study

| PCR assay | Sequence (5’-3’) | Ta | product size | purpose |
| --- | --- | --- | --- | --- |
| indel: AGC Kinase | F AAATGTTATCCATCGTGACCTC  R GATAGCCTTCTTTGGCTTCC | 52°C | 409bp | verification of indel |
| SNP: LRR | F TTGATTATGGGGCAACAGAAG  R TTGGCGAAGTCTTTCAAGAG | 51°C | 488bp | verification of SNP |
| deletion g6116_vir_ conventional PCR | F AGGATGTTTCAATTTCCTCGC  R CGGTTGTCCATTTTTCAAACAG | 52°C | 910bp | verification of g6116_vir_ deletion |
| deletion g6116_vir_ qPCR | F TTTCAATTTCCTCGCCGCC  R CGCAAGACCAGCCAATATAAAC  P HEX-TCGCCGCCGCCGCCTTCT-BHQ | na* | na |  |
| deletion g7085_vir_ conventional PCR | F GCGGGAAAACAAACGAAAC  R ATAGCCATTGGTCCTGGTC | 51°C | 894bp | verification of g7085_vir_ deletion |
| deletion g7085_vir_ qPCR | F ACATTCCCAACAAATGCCAG  R GTGGGAGACATTCATCAATGAC  P FAM-CCAACGAATACATCGAATGTACCTGATGACAA-BHQ1 | na | na |  |

*na= not applicable
